# Methoxylated Flavones from *Salvia Mirzayanii *Rech. f. and Esfand with Immunosuppressive Properties

**Published:** 2015

**Authors:** Abdul Majid Ayatollahi, Mustafa Ghanadian, Rahman Att-ur-Rahman, M. Ahmed Mesaik, Ahmad Shukralla Khalid, Fateme Adeli

**Affiliations:** a *Phytochemistry Research Center, Shahid Beheshti University of Medical Sciences, Tehran, Iran.*; b *Department of Pharmacognosy, School of Pharmacy, Shahid Beheshti University of Medical Sciences, Tehran, Iran.*; c *Isfahan Pharmaceutical Sciences Research Center, Isfahan Faculty of Pharmacy and Pharmaceutical Sciences, Isfahan University of Medical Sciences, Isfahan, Iran.*; d *International Center for Chemical and Biological Sciences H. E. J. Research Institute of Chemistry (ICCBS) University of Karachi, Karachi-75270, Pakistan.*

**Keywords:** Methoxylated flavones, *Salvia Mirzayanii*, Immunosuppressive, Oxidative burst, T-cell proliferation

## Abstract

Aerial parts of *Salvia Mirzayanii *was extracted with methanol. Methanol extract was suspended in water and defatted with petroleum ether. The defatted part was then partitioned between ethyl acetate and water. The ethyl acetate partition was chromatographed on a silica gel column to afford several fractions. Lymphocyte proliferation inhibitory assay of the resulted fractions was compared *in-vitro *and the fraction with more immunosuppressive activity was subjected to more purification to yield three methoxylated flavones: 5,7-dihydroxy,6,4’– dimethoxyflavone(1), 5-hydroxy,6,7,3’,4’–tetramethoxyflavone(2) and 5,3’-dihydroxy,6,7,4’– trimethoxyflavone (3).

Compounds 2 and 3 potently suppressed the proliferation of human blood lymphocytes with IC50 values of 1.3 ± 0.04 μg/mL and 1.3 ± 0.21 μg/mL in comparison with prednisolone as one of the lymphocyte suppressor drugs (IC50=1.45 ± 0.6 μg/mL). In phagocyte chemiluminescence assay, compounds 1 and 3 in peripheral mononuclear cells (PMNCs) exerted suppressive moderate activity against ROS with IC50 of 55.3 ± 0.4 and 36.2 ± 0.7 μg/mL, respectively, while compound 2 showed weak activity with IC50 values more than 100 μg/mL. In conclusion, compounds 2 and 3 have a similar suppressive effect more than compound 1 on PHA-activated lymphocyte proliferation, which might be because of their C-3’ oxidation pattern of ring B. It is indicated that the presence of 3’-OH or 3’-OMe in flavone ring B, caused more anti-proliferation activity than 3’-H. Oxidative burst assay showed more activity for compound 1 which is less methoxylated than others. It also showed more activity for compounds 3 than 2, which differ only in 3’-OH instead of 3’-OMe.

## Introduction

There are many reports from *Salvia* species to have a wide range of biological activities like antimicrobial, cytotoxic, and radical scavenging activities and are used in the folk medicine of different countries (1).* S. Mirzayanii *Rech. f. & Esfand (Labiateae family) with local name "Morr-e-Porzo" in the Iranian folk medicine is used as the antispasmodic or for the treatment of diabetes (2). In recent studies, methanol extract of *S. mirzayanii* showed lymphocyte proliferation suppression following reduction in the interleukin-2 in PHA activated cells (3). In a similar study, *S. mirzayanii* induced apoptotic effects in peripheral blood lymphocytes leading to reduction in lymphocyte proliferations and inhibition of immune responses (4). 

In a previous phytochemical study of this plant, Salvimirzacolide as a new sesterterpene is isolated from this plant (5). Essential oil of the plant was also analyzed by GC–MS. The main constituents were spathulenol, *δ*-cadinene, linalool, *α*-terpinyl acetate, *α*-cadinol, *β*-eudesmol, cubenol and linalyl acetate (6), out of which sesquiterpene compound, spathulenol has shown a weak inhibitory effect on lymphocyte proliferation (7). 

**Figure 1:**
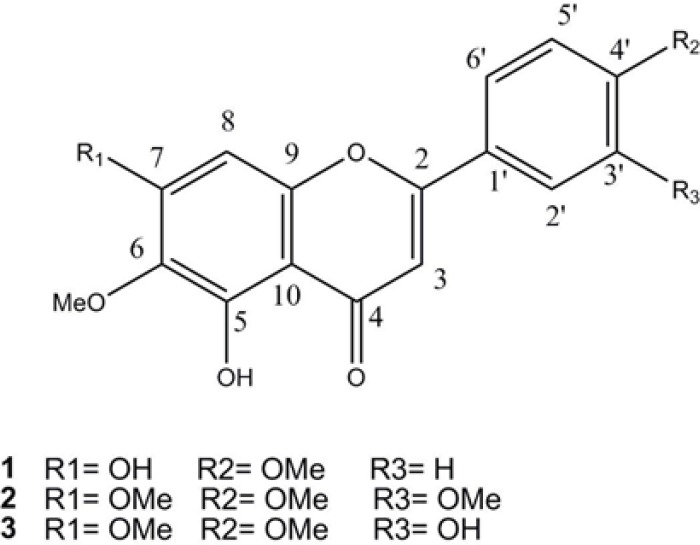
Structure of methoxylated flavones 1-3 from *Salvia Mirzayanii* Rech. f. & Esfand

Recently flavonoids show beneficial effects in the prevention of chronic diseases (8). In this research, locating for bioactive compounds responsible for the immunosuppressive activity of the plant, polymethoxylated flavones, with potent inhibitory effects on lymphocytes were isolated from the ethyl acetate fraction of the methanol extract (Figure 1). 

Methoxylated flavone derivatives could have various numbers of methoxyl groups on ring A or B. In recent studies, they showed anti-inflammatory, anti-malarial, anti-fungal, and anti-agonists to aryl hydrocarbon receptor in addition to apoptosis inducing properties (9). Methoxyflavones reported to have more chemopreventive activity than hydroxylated ones, including the reduction of the invasion of tumors in animal models, and suppressing cancer cell proliferations through proapoptoic properties (10). In one study on antiproliferative properties of methoxylated flavones on cancer cells, influence of ring A and B substituents on the activity were evaluated. In this study, methylation of the 6-OH increased the activity, and methylation of 7-OH and 3'-OH reduced the activity. It showed that more polar substitution patterns like 5, 7-dihydroxy, 5, 6, 7-trihydroxy, and 5, 7-dihydroxy-6-mehoxy at ring A, and 3',4'-dihydroxy at ring B could be important for antitumor activity (11).

## Experimental

*General*

Pure compounds were isolated by column chromatography using silica gel 60 (63–200 µm, Merck, Germany), MN-polyamide SC-6 (Macherey-Nagel, Germany) and thin layer chromatography (Silica gel 60 F254, Merck, Germany). The structure elucidations were done using different spectral methods, including 1H-NMR, 13C-NMR (BB and DEPT), and EI-MS. The NMR spectra were obtained with Bruker AV-500. The EI-MS spectra were taken with Varian MAT 312 spectrometer.

*Plant material*

*S. Mirzayanii* was collected from Kerman, Iran. It was identified by plant taxonomist in the Kerman Faculty of Pharmacy, Kerman University of Medical, Sciences, Kerman, Iran and a herbarium voucher specimen was deposited there. 

*Extraction and isolation*

The air dried plant material (2 Kg) was macerated in methanol (10 L) at room temperature for 3 days. The extraction was repeated three times. The extract was concentrated under reduced pressure to achieve a green gummy residue. Methanol extract was suspended in water and defatted with petroleum ether. The defatted part was then partitioned between ethyl acetate and water. The ethyl acetate fraction was chromatographed on normal phase silica gel column using gradient mixtures of dichloromethane: methanol (0 → 100%) to afford several fractions. Lymphocyte proliferation inhibitory assay of the resulted fractions was compared *in-vitro* on peripheral blood lymphocytes (12). The most active fraction with positive reaction with natural product reagent on its TLC profile was subjected separately to polyamide-SC6 column, using chloroform: methanol (0→20%) as the solvent system (13). Obtained fractions, were purified more by recycling HPLC using C-18 YMC column (MeOH/H_2_O, 7:3) to yield compounds 1-3 as the pure compounds. 

5,7-dihydroxy,6,4'–dimethoxyflavone (1)*. *Light yellow powder. ^1^H-NMR (500 MHz, DMSO-*d6*), δ_H_: 3.72, 3.91 (each 3H, *s*, OMe), 6.83 (1H, *s*, H-3) , 6.91 (1H, *s,* H-8), 6.93 (2H, *d*, *J* = 7.2 Hz, H-3', H-5'), 7.96 (2H, *d*, *J *= 7.2 HZ , H-2' ,6' ), 10.37 (7-OH), 12.91 (5-OH). ^13^C-NMR (125 MHz, DMSO-d6), δ_C_: 56.4 (4'-OMe), 59.9 (6-OMe), 91.5 (C-8), 102.6 (C-3), 105.0 (C-10), 115.9 (C-3',5'), 121.1 (C-4'), 128.4 (C-2',6'), 131.9 (C-6), 152.0 (C-9), 152.5 (C-5), 158.5 (C-7), 161.2 (C-4'), 164.0 (C-2), 182.1 (C-4). EI-MS *m/z* (%): 314 (100), 299 (86), 285 (21), 271 (32), 268 (20), 254 (5), 181 (18), 153 (37), 135 (5), 119 (14), 69 (15). 

5-hydroxy,6,7,3',4'–tetramethoxyflavone (2)*. *Light yellow powder. ^1^H-NMR (500 MHz, DMSO-*d6*), δ_H_:3.73, 3.85, 3.88, 3.93 (each 3H, *s*, OMe), 6.97 (1H, *s*, H-3) , 7.04 (1H, *s*, H-8), 7.13 (1H, *s*, *J* = 8.5 Hz, H-5'), 7.59 (1H, *d*, *J *= 2.0 HZ , H-2'), 7.72 (1H, *dd*, *J *= 8.5, 2.0 HZ , H-6'), 12.89 (5-OH). ^13^C-NMR (125 MHz, DMSO-*d6*), δ_C_:55.7 (7-OMe), 55.9 (3'-OMe), 56.4 (4'-OMe), 59.9 (6-OMe), 91.6 (C-8), 103.6 (C-3), 105.1 (C-10), 109.6 (C-2'), 111.7 (C-5'), 120.1 (C-6'), 122.8 (C-1'), 131.9 (C-6), 149.0 (C-3'), 152.0 (C-4'), 152.3 (C-9), 152.6 (C-5), 158.6 (C-7), 163.6 (C-2), 182.2 (C-4). EI-MS *m/z* (%): 358 (100), 357 (21), 343 (92), 329 (21), 315 (25), 312 (24), 181 (14), 163 (13), 153 (25), 148 (6), 136 (6), 69 (10). 

5,3'-dihydroxy,6,7,4'–trimethoxyflavone (3)*. *Light yellow powder. ^1^H-NMR (500 MHz, DMSO-*d6*), δ_H_:3.72, 3.86, 3.92 (each 3H, *s*, OMe), 6.81 (1H, *s*, H-3) , 6.90 (1H, *s*, H-8), 7.08 (1H, *d*, *J* = 8.5 Hz, H-5'), 7.47 (1H, *d*, *J *= 2.0 HZ , H-2'), 7.57 (1H, *dd*, *J *= 8.5, 2.0 HZ , H-6'), 9.42 (3'-OH), 12.89 (5-OH). ^13^C-NMR (125 MHz, DMSO-*d6*),δ_C_:55.8 (7-OMe), 56.4 (4'-OMe), 59.9 (6-OMe), 91.5 (C-8), 103.3 (C-3), 105.1 (C-10), 112.1 (C-5'), 113.1 (C-2'), 118.7 (C-6'), 122.9 (C-1'), 131.9 (C-6), 146.8 (C-3'), 151.2 (C-4'), 151.9 (C-9), 152.6 (C-5), 158.6 (C-7), 163.8 (C-2), 182.1 (C-4). EI-MS *m/z* (%): 344 (100), 343 (36), 329 (100), 315 (32), 301 (34), 298 (30), 181 (17), 153 (29), 149 (14). 

*Lymphocyte proliferation assay*

Human peripheral blood lymphocytes were incubated using three concentrations (0.5, 5, and 50 µg/mL) of the flavones 1-3 in triplicates in supplemented RPMI-1640 containing 5.0 µg/mL phytohemagglutinin (PHA) at 37 ºC in CO_2_ environment for about 72 hours. Incubation for more eighteen hours after the addition of [^3^H]-thymidine was done. Then cells were harvested with cell harvester and proliferation percent was determined by the radioactivity count as CPM reading by the Beta-scintillation counter (14).

*Phagocyte chemiluminscence assay*

The reactive oxidants (ROS formation) in whole blood during the phagocytosis oxidative burst processes were measured by the luminol-enhanced chemiluminescence assay procedure (14). Briefly, whole blood diluted in Hank's buffered salt solution was incubated with three concentrations (1, 10, and 100 μg/mL) of compounds 1-3 in triplicate for 30 minutes at 37 ºC. Then, 25 μL of 20 mg/mL Zymosan (Sigma Chemical Co, USA), followed by 25 μL of 0.07 mM luminol (Sigma Chemical Co., USA) were added to make a final volume of 100 µL. Positive and negative controls were included in the assay. The ROS chemiluminescence kinetic was monitored with a luminometer (Lab systems Luminoskan RS, Helsinki, Finland) for 50 minutes in the repeated scan mode. The peak and total integral chemiluminescence readings were recorded in the relative light unit (RLU).

## Results and Discussion

Compound 1 was isolated as a yellowish powder from ethyl acetate partition of the methanol extract of *Salvia Mirzayanii* with positive reaction to natural product reagent. NMR data (BB and DEPT) in addition to EI-MS molecular ion peak [M]^+ ^at *m/z* 314, indicated the molecular formula of C_17_H_14_O_6_ consistent with eleven degrees of unsaturation. EIMS spectrum showed *m/z *314 as a base ion together with fragment ions of *m/z *181 and 131, characteristic retro-Diels-Alder (RDA) fragments for a flavone-skeleton with two hydroxyl and one methoxyl groups on ring A and one methoxyl group on ring B (15,16). ^13^C-NMR showed one carbonyl carbon δc: 182.1 ppm, 14 aromatic carbons resonated between δc 91.5 to 164.0 ppm, and two methoxyl carbons δc:56.4 (4'-OMe), and 59.9 (6-OMe). The ^1^H-NMR spectrum showed AA'XX' spin pattern for H-2', 6' and H-3', 5' of ring B at δ_H_ 7.95 (*d*, *J* = 7.2 Hz, 2H, A and A' of AA'XX'), 6.92 (*d*, *J* = 7.2, 2H, X and X' of AA'XX'), two singlets, each one proton at δ_H_:6.91 (H-3) and 6.83 (H-8), in addition to two singlet signals each three protons related to the aryl methoxyl groups at δ_H_: 3.91, and 3.72 (s) and two deshielded protons at δ_H_: 12.91 and 10.37 ppm, which could be assigned as phenolic hydroxyl groups on flavone structure. Comparing the above data with published NMR data in the literature suggested the structure of compound 1 as 5,7-dihydroxy,6,4'–dimethoxyflavone (17). 

In a similar manner, compound 2 obtained as a yellowish solid with the molecular formula of C_19_H_18_O_7 _ based on ^13^C-NMR (BB and DEPT) and EI-MS molecular ion [M]^+ ^at *m/z* 358. The ^1^H-NMR spectrum showed ABX spin pattern similar to H-2', 6' and H-5' of ring B in flavones at δ_H_: 7.71 (*dd*, *J* = 6.8, 1.6 Hz, 1H, A of ABX), 7.59 (*d*, *J* = 1.6, 1H, and B of ABX), 7.13 (*d*, *J* = 6.8 Hz, 1H, X of ABX), two singlets at δ_H_:7.04 (H-3) and 6.97 (H-8), in addition to four singlet signals each integrated of three protons related to the aryl methoxyl groups at δ_H_: 3.93, 3.88, 3.85, and 3.73 (*s*) and one deshielded proton at δ_H_: 12.89 ppm, as 5-OH group (17). Therefore, based on NMR data, and published data in the literature, compound 2 was deduced to have the structure of 5-hydroxy,6,7,3',4'–tetramethoxyflavone (10, 18). 

Compound 3 was assigned the molecular formula C_18_H_16_O_7 _based on ^13^C-NMR and EI-MS molecular ion [M]^+^at *m/z* 344. NMR spectral data cleared that compound 3 and 2 were similar in structure differing only in 3'-OH instead of 3-OMe substitution. The ^1^H-NMR spectrum showed resonances at δ_H_: 7.57 (*dd*, *J* = 7.2, 2.0 Hz, 1H, H-2'), 7.46 (*d*, *J* = 2, 1H, H-6'), 7.10 (*d*, *J* = 7.2 Hz, 1H, H-5'), two singlets, each one proton at δ_H_:7.04 (H-3) and 6.97 (H-8), in addition to three methoxyl singlet signals at δ_H_: 3.92, 3.86, and 3.72 (*s*) and two deshielded protons at δ_H_ 12.89, and 9.41 ppm as 5-OH, and 3'-OH groups. Therefore, based on the above data and similar NMR data in the literature, compound 3 was determined as 5,3'-dihydroxy, 6,7,4'–trimethoxyflavone (19). Compounds 1-3 are displayed in Figure 1.

*Lymphocyte proliferation assay*

**Figure 2:**
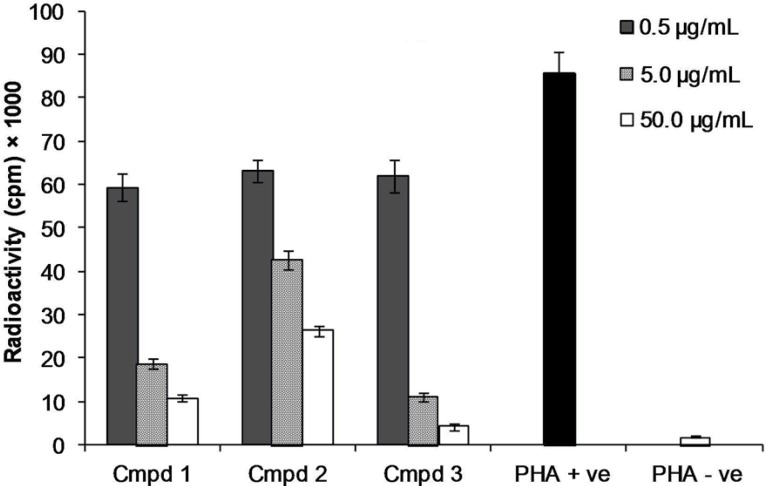
Effect of compound 1-3 on the proliferation of T-cells. T-cells were stimulated with PHA in the presence of three concentrations of compounds (0.5, 5.0 and 50.0 μg/mL) in three replicates. Phytohemagglutinin stimulated T-cells as positive control (PHA +ve) or unstimulated T-cells as negative control (PHA –ve) in the absence of the compounds were used as controls.

T-cells were activated by phytohemagglutinin mitogen in the presence of three concentrations (1, 10 and 100 μg/mL) of compounds 1-3. As shown in Figure 2, compounds 2 and 3 potently suppressed the proliferation of human blood lymphocytes dose dependently with IC_50_ values of 1.3 ± 0.04 μg/mL and 1.3 ± 0.21 μg/mL comparable with prednisolone as one of the lymphocyte suppressor drugs (IC_50_ = 1.45 ± 0.60 μg/mL). The results agreed with the immiunoinhibitory activity of other methoxlylated flavonoids like casticin from *Vitex agnus-castus* with potent suppressive effect on PHA stimulated T-cell (20).

*Effect on phagocyte oxidative burst and ROS production*

**Figure 3:**
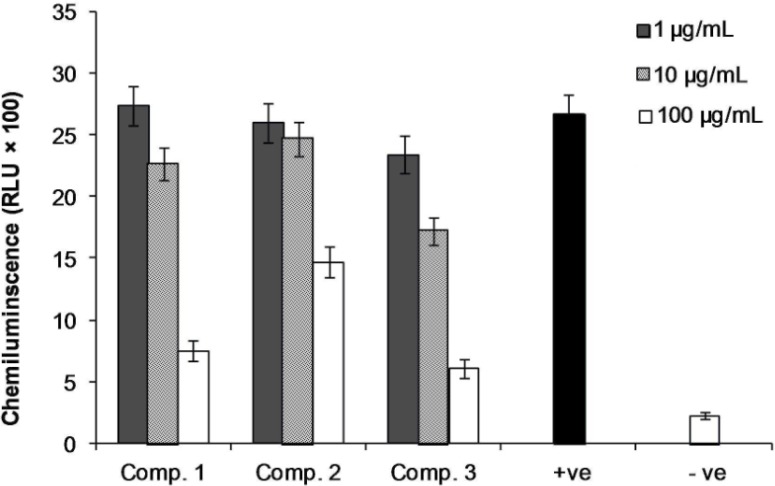
Effect of compound 1-3 on oxidative burst by phagocyte cells. Three different concentrations of compounds 1-3 (1, 10 and 100  g/mL) were incubated with human whole blood in triplicate and the chemiluminescence activity of phagocytic cells were measured by using serum opsonized zymosan and luminol. +Ve and –Ve controls are activated and non-activated cells, respectively, and both contain no compounds. Results are expressed as mean ± SD.

Compounds 1 and 3 on PMNCs in human whole blood, employing luminol and zymosan for the intracellular oxidative burst studies, exerted suppressive activities against ROS with IC_50 _values of 55.3 ± 0.4 and 36.2 ± 0.7 μg/mL, while compound 2 showed weak activity with IC_50_ values more than 100 μg/mL (Figure 3).

## Conclusion

Lymphocyte proliferation assay showed that compounds 2 and 3 have similar suppressive effects, but more than compound 1 on PHA-activated lymphocyte proliferation, which might be because of the C-3' oxidation pattern of ring B. It is indicated that the presence of 3'-OH or 3'-OMe in the flavones ring B, caused more anti-proliferation activity than 3'-H. It is agreed with another study on the structure activity requirements for methoxylated flavone cytotoxicity on human tumor cell lines. Oxidative burst assay showed more activity for compound 1, which is less methoxylated than others. It also showed more activity for compound 3 than 2, which differs only in 3'-OH instead of 3'-OMe. It means that 3'-methoxylation reduced phagocyte oxidative activity.
